# Physical exercise and cognition in older adults, a scientific approach scanty reported in Latin America and Caribbean populations

**DOI:** 10.3389/fspor.2024.1368593

**Published:** 2024-03-28

**Authors:** Alberto Jiménez-Maldonado, Iván Rentería, David K. Johnson, José Moncada-Jiménez, Patricia C. García-Suárez

**Affiliations:** ^1^ Facultad de Deportes, Universidad Autónoma de Baja California, Ensenada, México; ^2^ Department of Neurology, University of California, Davis, CA, United States; ^3^ Human Movement Sciences Research Center (CIMOHU), University of Costa Rica, San Jose, Costa Rica; ^4^ Department of Health, Sports and Exercise Sciences, University of Kansas, Lawrence, KS, United States

**Keywords:** physical exercise, general cognition, executive functions, older adults, Latin America

## Abstract

The advancement of public services, including the increased accessibility of health services, has led to a rise in life expectancy globally. As a result, aging populations are becoming more prevalent, raising concerns about cognitive decline. Fortunately, non-pharmacological methods, such as physical exercise, have been shown to mitigate the effects of aging on the brain. In this perspective article, we examined meta-analyses on the impact of physical exercise on cognition in older adults. The results indicate that combined exercise (i.e., aerobic plus strength training), has a significant positive effect on overall cognition and executive function. However, we found a lack of scientific studies on this topic in Latin American and Caribbean countries. Therefore, there is a pressing need for research to identify the feasibility of physical exercise interventions to improve cognitive skills in older adults from these regions.

## 1 Introduction

According to the World Health Organization (WHO) (1), life expectancy has increased since the past century, and by 2030, 1 in 6 individuals will be aged 60 or more, and epidemiological projections indicate that it is expected that the world will be inhabited by 2.1 billion older adults in 2050 (2). Nevertheless, the downside of this demographic transition in Latin America and Caribbean (LAC) countries is the heterogeneous and distributed social and health disparity-related risk factors for cognitive aging and dementia, a situation that is emphasized by the inadequate infrastructure and scarce scientific approaches to support the mental health in the individuals at the Caribbean Community (3). The growing elderly's demography emphasizes that health professionals should deeply understand older adults' biology and behavior pattern changes. In this sense, it is well known that morpho-functional changes in the brain accompany human aging (4). Some of these include a decline in the hippocampus volume (4), morphological changes in the cortex regions (neocortex areas) (5, 6), and changes in the brain metabolism (7) [see Matsson et al. (7) for an extensive review].

The age-related changes mentioned above enhance older adults' susceptibility to mental health conditions such as depression, anxiety, sleep disorders (6), and cognitive decline (4), being the last condition a precursor of a more severe disorder such as the Alzheimer's Disease (AD). The prevalence of AD in the general population of the United States in 2020 was 11.3% (i.e., approximately 6 million inhabitants) and 14.0% among Hispanics (i.e., approximately 0.71 million inhabitants) (8). The number of individuals in the general population with AD is expected to increase more than twice (i.e., approximately 13.85 million individuals) by 2060. It will be 423% higher among Hispanics (i.e., approximately 3.72 million inhabitants), yet the impact will be higher in adults older than 85, especially women (8).

Besides the natural aging process's impact on the human brain, voluntary movement has also become critical in protecting and maintaining cognitive function. It is known that older adults are among the most sedentary individuals, and higher sedentary time has been directly related to an increased risk of all-cause mortality in that population (9). Neuroscientists, exercise physiologists, and diverse health professionals have made significant efforts to identify pharmacological and non-pharmacological interventions focused on strengthening the brain's health in aging individuals. Owing to relevant scientific evidence, there is a consensus that physical exercise (PE) is a feasible method to attenuate aging-induced brain damage (10, 11). Some reports have identified that PE enhances the neuronal circuit in healthy individuals; therefore, the exercise benefits are not limited to a population with mental health disorders (12). Several randomized controlled trials have been the foundation for published meta-analyses (12–16), which analyzed in detail the impact of different exercise protocols on cognitive outcomes improvements in older adults, including variables such as general cognition, executive functions, and memory. These and other conditions are responsible for supporting real-life activities that allow older adults to orchestrate the complexities of daily tasks to plan actions, organize information, think abstractly, allocate mental resources, reason, solve novel problems, adapt to new situations, and act appropriately during social interactions (12–16). The current viewpoint article discusses the evidence reported in the meta-analyses about the impact of PE on cognitive control in older adults. At the end of the article, we present a brief reflection on how some variables hinder the rapid application of the evidence published in the countries of LAC.

## 2 Physical exercise on general cognition

**Figure 1:**
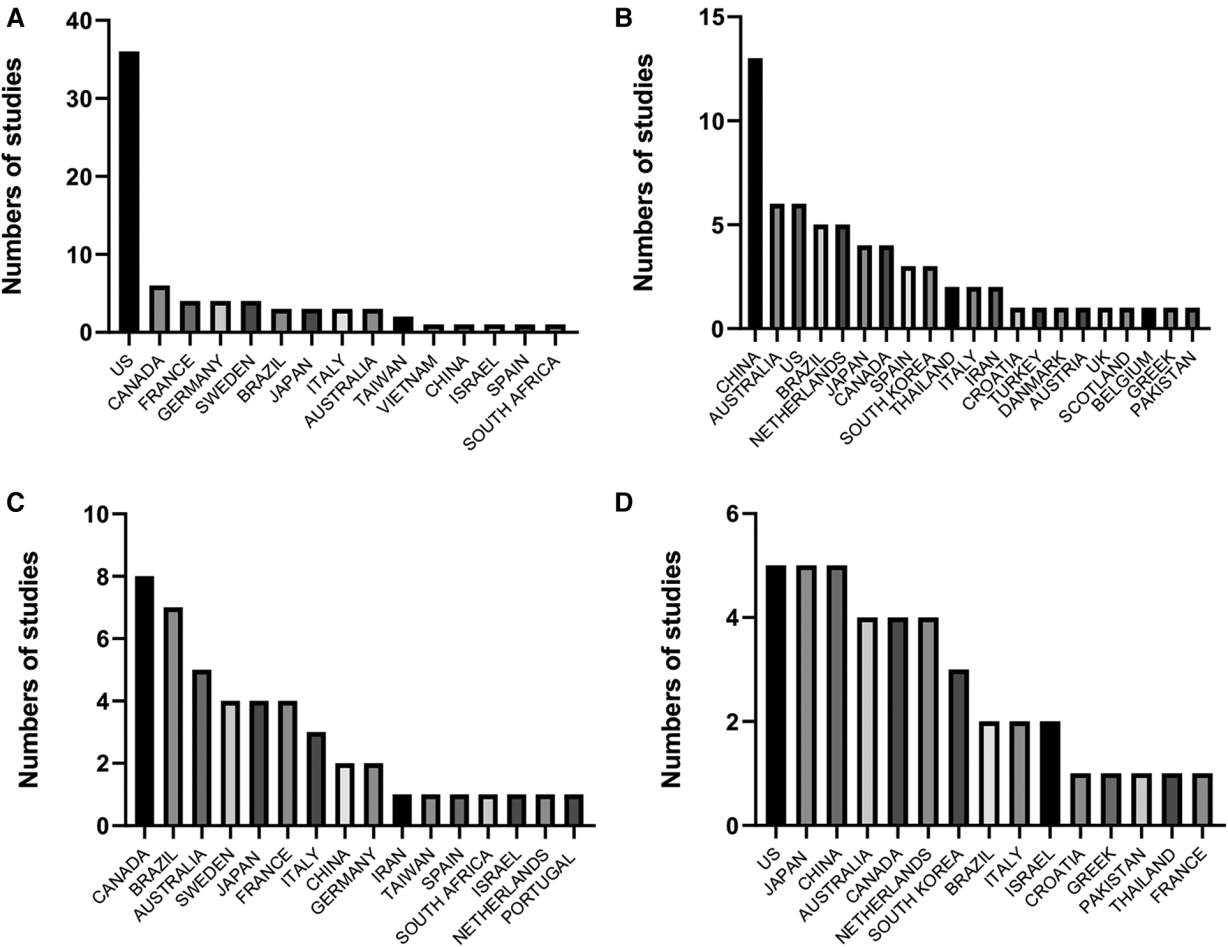
Frequency of studies included in meta-analyses on the effects of physical exercise on (**A**) general cognition in older adults without diseases, (**B**) general cognition in patients with mild cognitive impairment, (**C**) executive functions in older adults without diseases, and (**D**) executive functions in patients with mild cognitive impairment.

**Figure 2:**
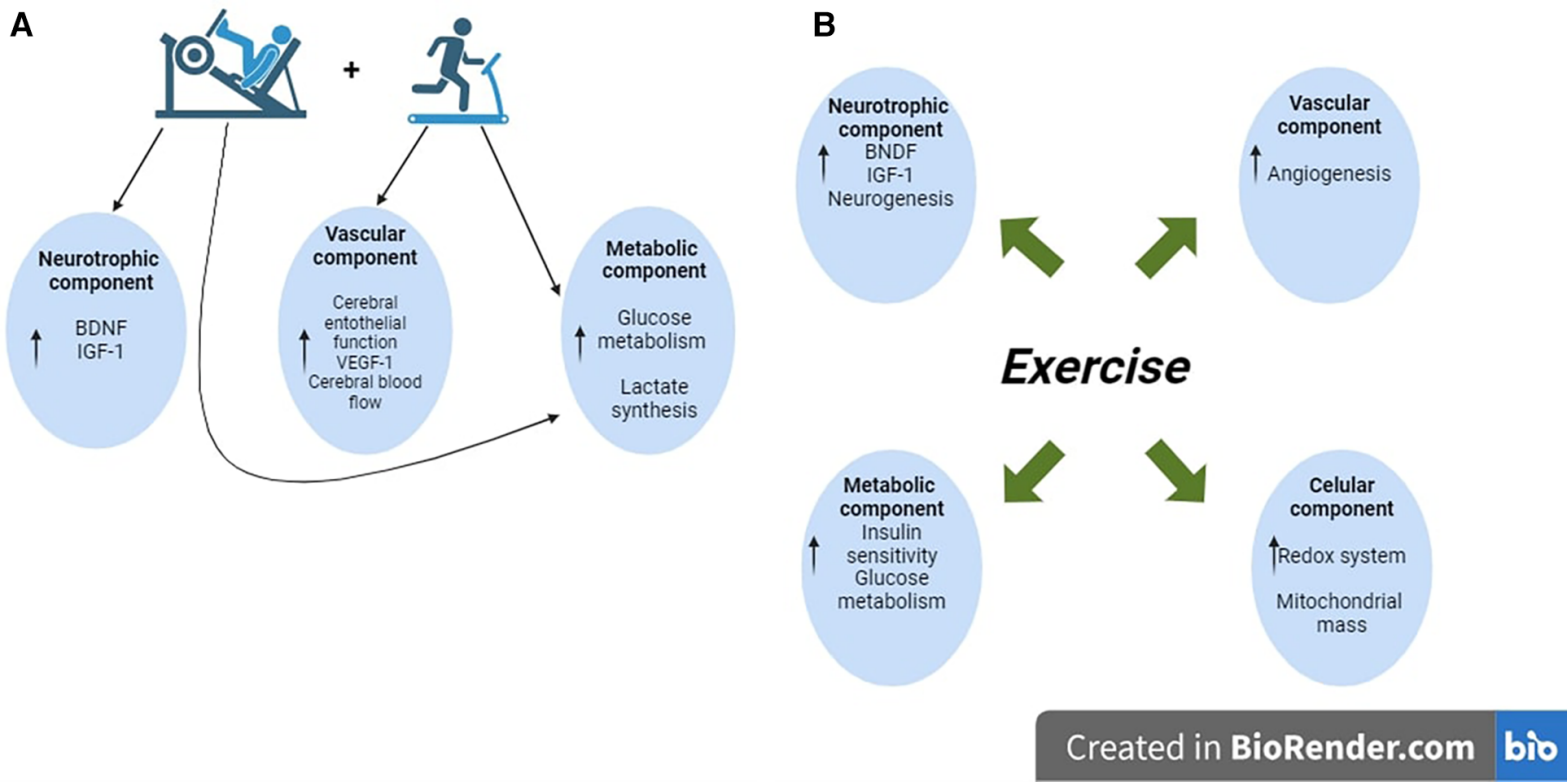
Schematic diagram showing the potential mechanism induced by the concurrent training to improve cognition in healthy older adults. (**A**) The available literature emphasizes that concurrent training is the best intervention to induce molecular and cellular adaptations to improve cognitive performance. Concretely, strength training increases the neurotrophic and lactate levels; those responses synergize with the effects of aerobic exercise, including improvements in cerebral blood flow. (**B**) In MCI, older adult patients do not identify the type of exercise intervention to induce better benefits on cognition. The reported findings indicate that physical exercise activates the neurotrophic component, increases mitochondrial mass and angiogenesis, and potentially improves glucose metabolism through insulin signaling.

Depending on the source, general cognition, also known as General Cognitive Ability (GCA) and defined as general mental ability, has been evaluated with valid and reliable instruments such as the Mini-Mental State Examination (MMSE) and the Montreal Cognitive Assessment (MoCA) (17). Meta-analytical evidence summarized in healthy older adults showed that studies implementing multicomponent interventions (i.e., protocols that included aerobic and strength exercises) were considered the most robust interventions for improving GCA (Figure 1A) (12–16). In the proposed molecular mechanism elicited by the multicomponent exercise interventions, the researchers highlighted neural growth factors (e.g., BDNF, IGF-1) as the main molecules synthesized during the resistance exercises and the regulators to induce more significant benefits in the brain compared with single aerobic interventions (12, 14). In addition, it was also suggested that the anaerobic resistance exercises (i.e., strength) increase the lactate concentration in the bloodstream; once in circulating blood, the lactate reaches the brain and enhances the expression of genes linked with cognition (e.g., BDNF) (18, 19). Those potential adaptations synergize with the well-identified effects induced by aerobic exercise, such as increased blood flow to the brain and upregulation of the brain's metabolism (20–22) (Figure 2A).

Together with the benefits of PE on brain functions in healthy older adults, the impact of exercise on general cognition in Mild Cognitive Impairment (MCI) patients has been another subject analyzed in published systematic reviews and meta-analyses (12, 15, 23–28). Figure 1B describes the origin of the individuals included in those studies. MCI is a condition that implicates severe cognitive decline compared with the aging effects *per se*; in this sense, it is known that MCI may continue until developing dementia and AD (29–32). The MCI (all-types) prevalence in LAC is 14.95%, values higher than those observed in high-income countries (e.g., United States and England) (33). Similar to other illnesses, several social and health factors increase the risk of developing MCI; the more commonly reported are education level, cardiometabolic diseases (i.e., hypertension, diabetes), vitamin D deficiency, and apolipoprotein E (APOE) e4 genotype (29, 32). In regard with this, was identified that LAC shows a socioeconomic environment, that increase substantially the risk to suffer MCI (33, 34). Considering MCI patients, and similar to healthy older adults, the protocols that include resistance exercises seem to be the most potent intervention to attenuate the disruption of the neuronal network linked with cognition (24, 27). Several potential mechanisms have been suggested to explain the positive effect of exercise that allows the recovery of the microenvironment in the brain, leading to attenuating the MCI progression. Some of these include the turn-up of trophic factors (e.g., BDNF, IGF-1), regulation of the redox system, increase in mitochondrial mass, angiogenesis, and neurogenesis; moreover, improved glucose metabolism was also reported (24, 35) (Figure 2B).

## 3 Physical exercise on executive functions

From another perspective, besides GCA, the effects of PE on executive functions (EFs) in older adults free from MCI have also been meta-analyzed (Figure 1C) (12–14, 36–38). The EFs are considered high-order cognitive processes unregulated by emotions and include selective attention, resistance to interference, working memory, mental flexibility, planning, verbal reasoning, and feedback utilization (39–42). It is known that the frontal lobe and parietal subcortical regions are activated during those tasks (43). The conclusions drawn from the current evidence are equivocal. For instance, a classical meta-analysis by Colcombe et al. (16) indicated that combined exercise interventions (i.e., aerobic exercises plus strength training) have a greater effect size on cognitive tasks, including EFs. The authors emphasized the dedifferentiation hypothesis to partially explain their findings (16). The hypothesis suggests that older adults recruit additional cortical areas to compensate for losses in neural efficiency.

On the contrary, other evidence that analyzed an aged population without diagnosed cognitive impairment found a non-significant effect of aerobic and resistance training on EFs (13). Indeed, the same study reported a significant effect of mind-body exercise (Tai Chi) on some EFs (i.e., working memory and processing speed). The authors emphasized mind-body exercise features to support their results; concretely, the researchers mentioned that the aerobic, strength, and flexibility exercises included in the Tai Chi movement sessions synergize to generate neuronal changes leading to improvements in the performance of cognitive activities, which included EFs (13). In agreement with the previous reports, others have also identified a significant effect of resistance, aerobic, and multicomponent training on EFs in healthy older adults ≥50 years (37). At this point, it is worth mentioning that throughout their analysis, the researchers found a pronounced benefit of resistance training on EFs (14, 37). In concordance with the above mentioned, a more recent meta-analysis concluded that PE is an effective intervention aimed at improving memory and EFs; in their study (12), the authors indicated that the BDNF synthesis, neocortical modifications, functional connectivity, and changes in the hippocampus volume as the principal adaptations to attain improvements in the cognitive performance.

On the other hand, the effects of PE on EFs in populations with MCI have also been recently meta-analyzed (Figure 1D) (12, 24, 27, 42). However, in this population, the findings are controversial; some authors reported null effects of PE (i.e., aerobic, strength, or multicomponent training programs) on EFs (12), on the contrary, recent evidence identified that resistance exercise induces the highest impact on the EFs compared with aerobic exercise (24, 44). The MCI has several molecular sources, which involve the accumulation of beta-amyloid plaques and neurofibrillary tangles (both molecular biomarkers of AD), glucose hypometabolism, cellular senescence, and neuronal atrophy (45–47). Subsequently, the PE interventions included in the meta-analytical evidence were likely insufficient (e.g., session duration, frequency of training, and exercise intensity) to induce molecular adaptations to improve the performance of EFs in MCI patients (12). Furthermore, it is worth mentioning that the MCI has subtypes; in general, those included amnestic MCI patients, who showed poor performance in episodic memory assessments, and non-amnestic MCI patients, who did not show fail in memory but displayed poor performance in other cognitive domains such as EFs, language, and visuospatial abilities (46). In the inclusion criteria of the articles, those characteristics were not considered (24, 44), which increased the risk of bias, leading to a high heterogeneity in the findings.

## 4 Physical exercise in the late adulthood in Latin America and Caribbean

Despite the promising results now communicated, unfortunately, the data reported in Figure 1 indicate that the LAC populations have been poorly studied; this situation hinders the direct applications of exercise recommendations as a non-pharmacological treatment to improve general cognition in older adults (10). In the following lines, we will emphasize some variables that could attenuate the benefits of PE on the brain functions (i.e., general cognition and EFs) in older adults from LAC countries. One reason is the education level; cross-sectional studies have constantly found a low education level in the older adult population from Mexico, Colombia, Costa Rica, and Brazil (48–51). On the contrary, in the United States, older adults show a higher education level than in LAC countries (52). In this sense, and knowing that it is a complex analysis, authors have mentioned that the education level is a moderator variable in the cognitive performance of older adults (53–56). Moreover, some reports indicated that education is more relevant for cognitive domains like memory (54). The prior information allows us to infer a lesser benefit of PE on the GCA in the older adults from LAC.

Moreover, the income differences among LAC countries and the world regions reported in Figure 1 are another factor that deserves attention. As it is well-known and in agreement with a recent World Bank (WB) report in 2023 (57), Mexico, Brazil, and Argentina show emerging markets and developing economies, while the United States, Canada, Australia, and Japan are targeted as advanced economies. These economic differences considerably impact the quality of life for the older adult population in each country. One of the potential disadvantages for the Latin American and Caribbean populations is their nutritional status. A recent report from the Food and Agriculture Organization (58) indicated that food insecurity is higher in LAC than in Asia, North America, and Europe (58). In this sense, current evidence indicates that the nutrition level is a determinant factor for brain functionality (59, 60). In light of this, it was suggested that a protein intake deficiency disturbs the antioxidant capacity, increasing the reactive oxygen species (ROS) levels and biomolecules that might potentially harm the brain (61, 62). Indeed, basic studies reported that a low-protein diet is linked with a decline in the neurotransmitter concentration associated with cognitive functions (63).

## 5 Conclusions

In regard with the meta-analyses articles analyzed, few studies from LAC have been carried out, however, we do not discard that the scarce evidences until now reported in the common database can be consequence by the language, all the articles analyzed were written in English language, we consider that systematic review and quantitative approach limited to Spanish, and Portuguese language are justified to identify the pool effect of PE on the cognition skills in LAC, with this activity, the barrier language could be cope. On the other hand, studies are also needed to clarify the participation of the variables above discussed in the responses to PE interventions and, consequently, to identify specific recommendations about the practice of PE to improve the brain function in older adults from LAC Countries. Finally, we emphasize that performing little PE is always better than none, and therefore, physical activity must be included in the lifestyle of older adults. This healthy habit will induce emotional and physiological benefits and potentially attenuate the Governments' health economic burden of countries classified as emerging and low-world economies.

## Data Availability

The original contributions presented in the study are included in the article/supplementary material, further inquiries can be directed to the corresponding author/s.
